# A study of the anti-inflammatory effect of the leaves of *Psidium guajava* Linn. on experimental animal models

**DOI:** 10.4103/0974-8490.72331

**Published:** 2010

**Authors:** Sarmistha Dutta, Swarnamoni Das

**Affiliations:** *Department of Pharmacology, Assam Medical College and Hospital, Dibrugarh – 786 002, Assam, India*

**Keywords:** Anti-inflammatory, arthritis, carrageenan, granuloma, *Psidium guajava*

## Abstract

**Introduction:**

The aim is to study the anti-inflammatory effect of the ethanolic extract of the leaves of ***Psidium guajava***(PGE) on experimental animal models.

**Materials and Methods:**

Fresh leaves were collected, air-dried, powdered, and percolated in 95% ethanol. Acute toxicity test was done according to OECD guidelines. Four groups of animals of either sex, weighing 150–200g of the species *Rattus norvegicus* were taken for the study (n = 6). Group A was taken as control (3% gum acacia in 10 mL/kg body weight), Group B as test group (PGE 250 mg/kg body weight), Group C as test group (PGE 500 mg/kg body weight), and Group D as standard (Aspirin 100 mg/kg body weight). The animals were studied for acute inflammation by Carrageenan-induced rat paw edema, subacute inflammation by Granuloma pouch method, and chronic inflammation by Freund’s adjuvant-induced arthritis method. Statistical analysis was done by one-way analysis of variance followed by multiple comparison tests.

**Results:**

In acute inflammation, there was significant inhibition of paw edema in Groups B, C, and D in comparison with Group A (**P** < 0.05). In subacute inflammation, there was significant inhibition of exudate formation in Groups B, C, and D in comparison to Group A (**P** < 0.05). In chronic inflammation, there was significant inhibition of paw edema and inhibition of weight reduction in Groups B, C, and D compared with Group A. Downregulation of arthritis index was also significant in Groups B, C, and D in comparison with Group A (*P* < 0.05).

**Conclusion:**

The ethanolic extract of PGE has significant anti-inflammatory activity.

## INTRODUCTION

The inflammatory process is the response to an injurious stimulus. It can be evoked by a wide variety of noxious agents (eg, infections, antibodies, or physical injuries).[1] The inflammatory response of the host is critical for interruption and resolution of the infectious process but also is often responsible for the signs and symptoms of disease. It involves a complex series of host responses, such as the complement, kinin, and coagulation pathways. An inability to kill or contain the microbe usually results in further damage due to progression of inflammation and infection.[2]

*Psidium guajava* Linn. (PGE), commonly known as Guava, belongs to the family Myrtaceae. It is a large evergreen or subdeciduous tree with smooth pinkish brown bark exfoliating in thin flakes. Leaves are opposite, 2–5.6 in long, oblong or elliptic oblong, and is faintly aromatic, lateral nerves prominent, petiole up to 0.3 in long.[3] Different parts of the plant are used in the indigenous system of medicine for the treatment of various human ailments, such as wounds, ulcers, bowels, and cholera.[4] It contains numerous compounds, such as polyphenolic compounds, flavonoids, tannins, ellagic acid, triterpenoids, guiajaverin, quercetin, and so on.[5] There is not much information regarding its anti-inflammatory activity. Considering this, the present study has been undertaken to evaluate the anti-inflammatory activity of the leaves of PGE on experimental animal models.

## MATERIALS AND METHODS

### Collection, identification, and extraction of plant materials

Fresh tender leaves of PGE, approximately 1 kg collected during April–May, 2009, were used for the study. The plant was authenticated by Dr. M. Islam, Professor of Life Science, Dibrugarh University, Assam, India. The plant material was air-dried at room temperature. The dried leaves were ground to fine powder and stored in air tight container.

### Preparation of the extract

An amount of 250 g of the dry powder obtained was soaked in 95% ethanol for 24 h in percolator. After 24 h it was allowed to percolate slowly and the extract was collected in Petri dishes.[6] The extract was concentrated in vacuum using rotary flash evaporator. There was a net yield of 22.6 g of concentrated extract (9.12%).

### Animals

The experiments were carried out in albino rats of the species *Rattus norvegicus* of either sex weighing 150–200 g. The animals were procured from Chakraborty Enterprise, Kolkata. The study was conducted in accordance with CPCSEA (Committee for the Purpose of Control and Supervision of Experiment on Animals) guidelines and the study was approved by the Institutional Animal Ethical Committee (Registration no.-634/02/a/CPCSEA). The animals were acclimatized for 1 week under laboratory conditions. They were fed with standard diet and water *ad libitum* was provided.

### Acute toxicity studies

Acute oral toxicity test for the ethanolic extract of leaves of PGE was carried out as per OECD guidelines 425.[7] Two arbitrary doses of 250 mg/kg and 500 mg/kg were selected for the study, as the extract was found safe even at doses more than 2000 mg/kg without any sign of toxicity or mortality.

### Anti-inflammatory studies

For each experiment, the animals were divided into 4 groups with 6 animals in each group.

- Group-A (control) received 3% gum acacia 10 ml/kg p.o.
- Group-B (Test-1) received PGE leaf extract 250 mg/kg p.o.
- Group-C (Test-2) received PGE leaf extract 500 mg/kg p.o.
- Group-D (standard) received aspirin 100 mg/kg p.o.

All the drugs were administered orally and the volume of medicaments kept constant at 10 mL/kg body weight of the animals.

#### (a) Anti-inflammatory Study Against Acute Inflammation

The anti-inflammatory activity of ethanolic extract of leaves of PGE against acute inflammation was tested by carrageenan-induced rat paw edema method.[8] Acute inflammation was produced by subplantar injection of 0.1 mL of freshly prepared 1% carrageenan suspension in normal saline in the left hind paw of rats in each group.[9] The animals were treated with 3% gum acacia, PGE, or aspirin in the respective groups, 1 h before carrageenan injection.[8] The paw volume was measured plethysmometrically as described by Chattopadhyay *et al*.[10] just before carrageenan injection, that is, at “0” h and then at 1^st^, 2^nd^, 3^rd^, and 4^th^ h after carrageenan injection. Increase in paw edema was measured as the difference between the paw volume at “0” h and paw volume at the respective hour. The percentage inhibition of the rat paw edema was calculated after each hour of carrageenan injection up to 4 h by the formula described by Sudjarwo Agus.[11]

$$\%\;\mathrm{Inhibition}\frac{\left(\mathrm{Control}\;\mathrm{mean}\;-\;\mathrm{treated}\;\mathrm{mean}\right)}{\mathrm{Control}\;\mathrm{mean}}\times 100$$

The data were subjected to statistical analysis using one-way analysis of variance (ANOVA) followed by Dunnet’s multiple comparison test. *P* values < 0.05 were considered significant.

#### (b) Anti-inflammatory Study Against Subacute Inflammation

The anti-inflammatory activity of ethanolic extract of PGE leaves against subacute inflammation was tested by Granuloma pouch method.[12] Rats were anesthetized with ether, and subcutaneous dorsal air pouches were then prepared at the backs, by injecting 20 mL of air with the help of a fine needle, after proper shaving and disinfection. Then 1 mL of 20% carrageenan suspension in sesame oil was injected into each pouch; 48 h later air was withdrawn from the pouch and 72 h later any resulting adhesions were broken. The animals were treated with 3% gum acacia, PGE, or aspirin in the respective groups for 4 days starting from the day of pouch formation. On the 5th day, the animals were sacrificed under ether anesthesia. The pouch was opened and exudates were sucked out and the amount measured in glass cylinders. The average values of the exudates of the control and the test groups were calculated. The percentage inhibition was then calculated for all the groups.[12]

#### (c) Anti-inflammatory Study Against Chronic Inflammation

The anti-inflammatory activity of ethanolic extract of PGE against chronic inflammation was tested by adjuvant-induced arthritis method in rats.[13]

On day 1, the animals were injected into the subplantar region of the left hind paw with 0.1 mL of complete Freund’s adjuvant. Dosing with the test compounds or the standards to the respective groups was started on the same day and continued for 12 days. The paw volumes of both sides and the body weights were recorded on the day of injection. The paw volumes were measured plethysmographically as in the paw edema test. On day 5, the volume of the injected paw was measured again, indicating the primary lesion and the influence of the therapeutic agent on this phase. The severity of the induced adjuvant disease was followed by measurement of the noninjected paw (secondary lesions) with a plethysmometer. Purposely, from day 13 to 21, the animals were not dosed with the test compound or the standard. On day 21, the noninjected paw volume and the body weight were determined again[14] and the polyarthritis severity was graded on a scale of 0–4: 0 = no swelling; 1 = isolated phalanx joint involvement; 2 = involvement of phalanx joint and digits; 3 = involvement of the entire region down to the ankle; 4 = involvement of entire paw, including ankle. The maximum joint score was 12, including 3 secondary arthritis paw for each rat. 14

1. For primary lesions : The percentage inhibition of paw volume of the injected left paw over control was measured at day 5.
2. For secondary lesions : The percentage inhibition of paw volume of noninjected right paw over control was measured at day 21.
3. An Arthritis index was calculated as the sum of the scores as indicated above for each animal.[13]

### Statistical analysis

For all the above methods, the results were expressed as mean ± standard error of mean. Statistical analysis was done using one-way ANOVA followed by Dunnet’s multiple comparison test. *P* values < 0.05 was considered significant.

## RESULTS

As seen from the above study, in the carrageenan-induced rat paw edema for acute inflammation, the standard drug (aspirin 100 mg/kg) showed maximum inhibition at the end of the 1^st^, 2^nd^, 3^rd^, and 4^th^ h. The inhibition of paw edema was maximum at the end of 3r h for all the drugs and the effect of PGE at a dose of 500 mg/kg was better than that of PGE at a dose of 250 mg/kg when given orally. There is no significant difference of inhibition of paw edema between PGE at a dose 500 mg/kg and the standard group at the end of 3^rd^ and 4^th^ h [Table 1].

**Table 1:** Anti-inflammatory activity of the ethanolic extract of *Psidium guajava* leaves on carrageenaninduced rat paw edema in albino rats

Group	Drug dose p.o.	Mean increase in paw volume (Mean ± SEM) (mL)(% Inhibition within parentheses)			
		1st h	2nd h	3rd h	4th h
A (Control)	10 mL/kg	0.20 ± 0.02	0.28 ± 0.01	0.54 ± 0.04	0.30 ± 0.05
B (PGE)	250 mg/kg	0.15 ± 0.04^a^,^b^	0.20 ± 0.01^a^,^b^	0.34 ± 0.08^a^,^b^	0.19 ± 0.07^a^,^b^
		(25.00%)	(28.57%)	(38.46%)	(36.66%)
C (PGE)	500 mg/kg	0.11 ± 0.01^a^,^b^	0.13 ± 0.06^a^,^b^	0.23 ± 0.04^a^,^c^	0.13 ± 0.01^a^,^c^
		(45.00%)	(53.57%)	(58.82%)	(56.66%)
D (Aspirin)	100 mg/kg	0.09 ± 0.01^a^	0.11 ± 0.07^a^	0.20 ± 0.02^a^	0.11 ± 0.01^a^
		(55.21%)	(60.71%)	(64.70%)	(62.12%)
	F	285	400.4	175.9	355.9
One-way ANOVA	df	20,3	20,3	20,3	20,3
	P	<0.05	<0.05	<0.05	<0.05

PGE, *Psidium guajava*; SEM, standard error of mean; ANOVA, analysis of variance, Values = Mean±SEM, n = 6 in each group;

a → P < 0.05 when compared with control;

b → P < 0.05 when compared with standard;

c → P > 0.05 when compared with standard; ANOVA followed by Dunnet’s multiple comparison test.

In the Granuloma pouch method for subacute inflammation, the inhibition of exudate formation was maximum for the standard drug (aspirin 100 mg/kg). It was observed that PGE produced highly significant inhibition of exudate formation in comparison with the control but significantly less than that of the standard in both the doses. The test drug showed dose-dependent inhibition of the exudate formation and the effect of PGE at the dose of 500 mg/kg was better than that of PGE at the dose of 250 mg/kg when given orally [Table 2].

**Table 2:** Anti-inflammatory activity of the ethanolic extract of Psidium guajava leaves on subacute inflammation by granuloma pouch method in albino rats

Group	Drug dose p.o.	Mean volume of exudate (Mean ± SEM) (mL)	Inhibition of exudate formation (%)
A (Control)	10 mL/kg	3.60 ± 0.04	-
B (PGE)	250 mg/kg	2.20 ± 0.25^a^,^b^	38.88
C (PGE)	500 mg/kg	1.30 ± 0.03^a^,^b^	63.88
D (Aspirin)	100 mg/kg	1.05 ± 0.02^a^	70.83
	F	936.9	
One-way ANOVA	df	20,3	
	P		>0.05

PGE, *Psidium guajava*; SEM, standard error of mean; ANOVA, analysis of variance, Values = Mean±SEM, n = 6;

a → P < 0.05 when compared with control;

b → P < 0.05 when compared with standard; ANOVA followed by Dunnet’s multiple comparison test.

In the Freund’s complete adjuvant-induced arthritis method for chronic inflammation, it was observed that for primary lesions aspirin 100 mg/kg showed maximum inhibition of paw volume of the injected left paw. PGE at the doses of 250 and 500 mg/kg produced significant dose-dependent effect as compared with control. However, the effect of PGE at both the doses was significantly less than that of aspirin 100 mg/kg. For secondary lesions, the percentage inhibition of paw volume of noninjected right paw over control was maximum for the standard drug (aspirin 100 mg/kg).The test drugs at both the doses showed significant inhibition of paw volume as compared with control, which was dose dependent. There is no significant difference between PGE at the dose of 500 mg/kg and the standard. Arthritis index was calculated on 21^st^ day of adjuvant injection. Arthritis index was highest in the control group and lowest for the standard. Both the test groups and the standard group significantly downregulated the arthritis index in the adjuvant-induced arthritis rats as compared with the control group. To measure the change in weight, all the animals were weighed on the 1st day and again on the 21 ^st^ day. PGE at the dose of 250 and 500 mg/kg produced dose-dependent reduction in decrease of weight, both of which were significant as compared with the control. The standard group (aspirin 100 mg/kg), showed highest reduction in decrease of weight [Table 3].

**Table 3:** Anti-inflammatory activity of the ethanolic extract of *Psidium guajava* leaves on carrageenaninduced rat paw edema in albino rats

Group	Drug dose p.o.	Increase in paw volume in mL (% inhibition in parentheses)		Weight change on 21^st^ day (% change in parentheses)	Arthritis index
		On 5^th^ day	On 21^st^ day		
A (Control)	10 mL/kg	0.92 ±0.02	0.52 ± 0.01	-28 0.32	7.8 0.24
B (PGE)	250 mg/kg	0.74±0.03^A^,^B^	0.20±0.02^a^,^b^	-15 ± 0.21^a^,^b^	5.9 ± 0.16^A^,^B^
		(20.12%)	(62.14%)	(46.42%)	
C (PGE)	500 mg/kg	0.67±0.12^a^,^b^	0.140.04^a^,^c^	-10 0.3^a^,^b^	4.5 0.14^a^,^b^
		(28.04%)	(74.18%)	(64.28%)	
D (Aspirin)	100 mg/kg	0.56 0.02^a^	0.11±0.02^a^	-5 0.14^a^	3.2 0.10^a^
		(39.18%)	(80.67%)	(82.14%)	
One-way	F	182.8	82.36	66.80	96.32
ANOVA	df	20,3	20,3	20,3	20,3
	P	<0.05	<0.05	<0.05	<0.05

PGE, *Psidium guajava;* SEM, standard error of mean; ANOVA, analysis of variance, Values = Mean±SEM, n = 6;

a → P < 0.05 when compared with control;

b → P < 0.05 and

c → P > 0.05 when compared with standard; ANOVA followed by Dunnet’s multiple comparison test in albino rats.

## DISCUSSION

Carrageenan-induced paw edema is a biphasic response. The first phase was mediated through the release of histamine, serotonin, and kinins and the second phase was due to release of prostaglandin-like substances in 2–3 h.[15] Drugs that inhibit carrageenan-induced paw edema may act through inhibition of leukocyte migration and prostaglandin synthesis.[16] In the present study, maximum paw edema was observed at the end of 3^rd^ h of carrageenan injection, that is, after the release of all these mediators of inflammation. The probable cause of anti-inflammatory action against acute inflammation might be due to the inhibition of some or all of the mediators released within 3 h of carrageenan injection.

Subacute inflammation involves infiltration of macrophages, neutrophils, and proliferation of fibroblasts.[17] Flavonoids found in the plant extracts have antiproliferative activity, which is found to cause a decrease in the weight and volume of contents of granuloma.[18] Also cytokines are found to be the important mediators in the formation and maintenance of granuloma.[19] So it is speculated that the anti-inflammatory activity against subacute inflammation by PGE may be due to flavonoids found in the extract.[20]

Freund’s adjuvant-induced arthritis is a widely used chronic model for inflammation. One of the reasons for the wide utilization of this model is due to the strong correlation between the efficacy of therapeutic agents in this model and in rheumatoid arthritis in humans and it is characterized by very rapid erosive disease.[21] Adjuvant arthritis is characterized by weight loss[22] and body weight loss is associated with increased production of proinflammatory cytokines, such as tumor necrosis growth factor-α (TNF-α) and interleukin-1 (IL-1).[23] These cytokines have profound effects on the hormones that govern metabolism and also act directly on the metabolic target organs, such as muscle, liver, gut, and brain.[24] The result is an increase in resting energy expenditure, a net export of amino acids from muscle to liver, an increase in gluconeogenesis and a marked shift in liver protein synthesis away from albumin and toward production of acute phase proteins, such as fibrinogen and C-reactive protein.[25] Thus the arthritis-induced reduction in weight can be prevented by the test drug and it may be due to inhibition of TNF-α and IL-1. The leaves of guava are rich in flavonoids, in particular, quercetin. Much of guava’s therapeutic activity is attributed to these flavonoids. The flavonoids have demonstrated anti-inflammatory activity.[26] Also flavonoids have antiproliferative activity, which is found to cause a decrease in the weight and volume of contents of granuloma in inflammation.[27]

The present study demonstrated that the ethanolic extract of the leaves of PGE showed significant anti-inflammatory activity against acute, subacute, and chronic inflammation. However, further studies are required to establish and elaborate the molecular mechanism for proper clinical utility. Also, the development of more purified products of PGE for the treatment of various inflammatory diseases should be encouraged.
